# Exploring Prenatal Care Quality and Access During the COVID-19 Pandemic Among Pregnant Immigrants in Philadelphia Through the Lens of Community-Based Organizations

**DOI:** 10.1089/whr.2022.0112

**Published:** 2023-05-17

**Authors:** Deanna Marshall, Mikaela Perez, Xi Wang, Meredith Matone, Diana Montoya-Williams

**Affiliations:** ^1^ PolicyLab, Children's Hospital of Philadelphia, Philadelphia, Pennsylvania, USA.; ^2^ Department of Epidemiology & Biostatistics, Dornsife School of Public Health, Drexel University, Philadelphia, Pennsylvania, USA.; ^3^ Department of Pediatrics, Perelman School of Medicine, University of Pennsylvania, Philadelphia, Pennsylvania, USA.; ^4^ Department of Neonatology, Children's Hospital of Philadelphia, Philadelphia, Pennsylvania, USA.

**Keywords:** health care access, health inequalities, health services research, immigrant health, Pennsylvania, prenatal care, undocumented immigrants

## Abstract

**Background:**

The peak of the COVID-19 pandemic led to decreased maternal and child health care engagement, especially among marginalized populations. Existing disparities in prenatal care access and quality faced by pregnant immigrant people are likely to be amplified by the pandemic.

**Materials and Methods:**

We conducted a study with direct service providers (DSPs) at community-based organizations (CBOs) serving pregnant immigrant families in the Philadelphia region. Semistructured interviews addressed barriers and facilitators to prenatal health care access and engagement among immigrant families both before and then after the onset of the pandemic in March 2020. Additional questions elicited context about the demographics of service populations, organizational connectedness to health care providers, and pandemic-related operational changes.

**Results:**

Between June and November 2021, 10 interviews were conducted in English and Spanish with DSPs at 5 CBOs. Primary themes included diminished access and quality of care received due to decreased language accessibility, increased restrictions around support persons, shifts to telemedicine, and changes to appointment scheduling. Additional themes included heightened hesitancy engaging with services due to documentation status, confusion around legal rights, financial strain, and health insurance status. Interviewees provided suggestions for improving service access during and postpandemic for immigrant pregnant people, including implementation of culturally responsive group prenatal care, institutional policies to improve understanding of legal rights, and increased financial supports.

**Conclusions:**

Understanding emergent and exacerbated barriers to prenatal care access and quality during the COVID-19 pandemic provides context for how to improve health equity for immigrant pregnant people through public health and health care policies as the pandemic continues, and once it has subsided.

## Introduction

Research has shown that pregnant immigrant people face significant interrelated challenges navigating the health system, including lack of knowledge of existing resources, language barriers, and discrimination, and for those who are undocumented, fear of deportation.^1^ Prenatal care is an integral and basic health service recommended by the American College of Obstetricians and Gynecologists for all pregnant people; however, foreign-born women have much lower rates of prenatal care utilization than US-born women.^2,3^ Each year, ∼25% of births in the United States are to foreign-born mothers and 7.5% of births are to undocumented immigrant people who are far less likely to have access to quality prenatal care than other US residents.^2,4,5^

The last four decades have witnessed an increase in punitive immigration policies, including the widely publicized changes to the federal public charge rule in 2019, which greatly expanded the reasons immigrants might be denied legal residence or face deportation.^6,7^ Although the expanded rule was rescinded in 2021, research suggests this and other recent policies led many immigrants in the United States to avoid health and social benefits due to fear and confusion.^7^

This shifting federal policy landscape disincentived health care seeking among immigrant individuals.^8,9^ The emergence of the COVID-19 pandemic further threatened pregnant immigrant people's already fragile access to healthcare. Undocumented people were disproportionately affected by the COVID-19 pandemic. This occurred by their exclusion from federally funded programs such as the CARES Act,^10–12^ but also because this population was at higher risk for contracting COVID-19 and suffering from severe illness due to sociostructural factors related to type of employment, housing conditions, insurance eligibility, and language barriers to timely public health messaging. Pregnant immigrant people were not immune to this excess risk, as evidenced by associations between increased risk of SARS-CoV-2 infection and limited English proficiency in this population.^13–15^

Despite the COVID-related health disparities affecting immigrant communities, most existing research only highlights the risk factors that could lead to diminished health care access and outcomes, rather than elucidating the experiences of families during the pandemic. In this study, we sought to assess the lived experiences of immigrant pregnant people accessing prenatal care during the pandemic through the lens of direct service providers (DSPs) from immigrant-focused community-based organizations (CBOs). Previous research has documented that trusted CBOs serve as safety nets for immigrant communities, especially for individuals barred from insurance for economic or documentation reasons.^16,17^ Such organizations are instrumental in disseminating public health information and assisting immigrant communities in accessing both health care and health insurance for those eligible.^17,18^

Throughout the COVID-19 pandemic, immigrant-focused CBOs have additionally played critical roles in increasing access to COVID-19 testing and vaccination for immigrant communities.^19,20^ Given the role that DSPs often play for their immigrant clients, such as serving as trusted sources of information, intermediaries, or care navigators, they are well situated to discuss the mechanisms by which the pandemic, and its associated health care and public health policies, has impacted existing disparities for pregnant immigrant people. Critically, we felt this approach allowed us to gather information that might be challenging or uncomfortable for immigrant people to disclose themselves, due to immigration status-related fears or the inherent power imbalances that might be felt by immigrant research participants.^21^

Thus, in this qualitative study, we interviewed a sample of DSPs serving pregnant people through CBOs, whose work focuses on diverse immigrant groups. Our goal was to highlight multilevel challenges and facilitators to prenatal care access among pregnant immigrant people to inform efforts that seek to remedy recent exacerbations in disparities in health care access and outcomes among this community. We chose to focus on access to prenatal care among immigrant people for two reasons. First, pregnancy has significant health implications and risks for birthing people, which can be addressed during routine prenatal visits.^22^ Second, health care access and outcomes during pregnancy are intimately related to infant health.^23^ Thus, understanding access to quality prenatal care for immigrant populations has important transgenerational public health implications.

## Materials and Methods

### Study participants

This cross-sectional qualitative study included a sample of DSPs at CBOs serving pregnant immigrants in the Philadelphia region. Philadelphia is a large metropolitan region that has been considered an immigrant gateway city in recent decades, given the rapidly increasing numbers of immigrants settling there.^24^ As such, this city represents an optimal setting in which to study health care access among both established and recently arrived immigrants. DSPs were eligible to participate in the study if they worked directly with pregnant immigrants both before and after March 2020, the onset of the COVID-19 pandemic in the Philadelphia region, and until the time of their recruitment to this study. This time period was chosen so that they could speak about clients' experiences both before and after the onset of the pandemic.

All study participants were employees of CBOs in Philadelphia, which serve immigrant communities from a variety of world regions and ethnic backgrounds. Examples of DSP roles within their respective CBOs were support persons, social workers, case workers, and health care navigators. This study population was chosen because these individuals serve as informed close confidants of pregnant immigrant people and have insight into local barriers and sources of resilience for pregnant immigrant people. DSP participants were recruited through virtual study flyers in English and Spanish and contact between the study team and leadership at CBOs who shared the opportunity with their staff. Participants were compensated for their time with a $50 gift card.

### Data collection

The study team conducted in-depth, semistructured qualitative interviews with eligible participants between June and November 2021. Interview guides (Supplementary Data S1) were created in English and then translated into Spanish by bilingual research team members. Interviews were conducted in Spanish (by a bilingual team member) or English, depending on the preference of the interviewee. Interviews lasted between 50 and 75 minutes and were audio-recorded, transcribed, and reviewed for accuracy. Participants provided informed consent before participating.

### Measures

The primary aim of this qualitative work was to identify barriers and facilitators to accessing and engaging with quality prenatal care services during the COVID-19 pandemic among pregnant immigrants. To provide context for COVID-related prenatal experiences, we first asked DSPs about the “typical” prenatal care experience for their clients before the onset of the pandemic. We then asked them to describe what they had seen and learned about their clients' experiences accessing prenatal care after March 2020. Additional questions elicited context about the demographics of service populations, organizational connectedness to health care providers, and pandemic-related operational changes. A secondary aim was to gather suggestions for improving prenatal care access and quality for pregnant immigrants as the pandemic continues, or eventually subsides. Thus, we ended interviews by asking interviewees to suggest changes that could be made to prenatal health care access, which would improve health care experiences for pregnant immigrants.

### Analysis

Audio-recorded interviews were transcribed verbatim in the language in which they were conducted. The team used NVivo 12 software for coding and analysis of de-identified transcripts. The study team then utilized a subset of four interview transcripts to create a codebook using an inductive approach, allowing themes to arise from the qualitative interview content. Two members of the study team (D.M. and M.P.) coded transcripts using constant comparison to ensure intercoder reliability and to refine code definitions and application.

Specific themes were first individually coded and then grouped together into codes representing broader themes, creating codes and subcodes. Group consensus was utilized to resolve coding discrepancies. We then conducted a thematic analysis of these data. Subsequently, the other members of the team provided feedback on the organization of themes in an iterative manner. Thematic saturation was reached with 10 participants when no novel theme arose after coding the last two interviews. There was no large enough sample of Spanish language transcripts to determine thematic differences between DSPs who requested that interviews were conducted in Spanish.

This study was deemed exempt from institutional review board review after being submitted to the Committee for the Protection of Human Subjects at Children's Hospital of Philadelphia.

## Results

Between June and November 2021, we interviewed 10 DSPs across 5 immigrant-serving CBOs in the Philadelphia region. Of those interviews, 8 were conducted in English and 2 in Spanish. DSPs reported serving diverse immigrant communities, including clients who emigrated from Africa, Asia, the Caribbean, Central America, and South America. The client population served is a mix of documented and undocumented individuals, both with and without health insurance. We asked DSPs questions about their clients' experiences both before, and then after the onset of the COVID-19 pandemic regarding pregnancy, prenatal care access, and quality of care provided.

### Pre-COVID experiences with prenatal care

Before the pandemic, DSPs reported that their clients most often went to Federally Qualified Health Centers (FQHCs) run by the city for prenatal care, rather than hospital-based clinics, because of the perceived lower cost and options for those without insurance. Philadelphia FQHCs run by the city provide health services regardless of citizenship or documentation status, if the person resides in Philadelphia, making them the default option for undocumented pregnant immigrants within the city.

Table 1 lists facilitators and barriers to prenatal care access that DSPs discussed pregnant immigrants were experiencing before the onset of the pandemic, along with exemplary quotes. With respect to barriers, two major themes arose. DSPs discussed the language barriers their clients faced not only when making appointments but also during visits. They also spoke of the challenges associated with using *ad hoc* interpreters, especially when children serve as interpreters, given that children are not equipped to accurately relay medical information to patients. The second barrier DSPs highlighted was related to their clients' lack of knowledge of benefits they were eligible for as immigrants.

**Table 1.** Immigrants' Access to and Experiences with Prenatal Care Before the Onset of the COVID-19 pandemic as reported by Direct Service Providers

Barriers to receiving high-quality prenatal care	
Inconsistent access to language-concordant care (*i.e.*, care in preferred language)	“I ask them, ‘What did the doctor say?’ And they say, ‘I really don't remember, because they had a translator, and it was difficult to me. And I really don't know. I know she said everything is okay. But then, no more than that. No more.’ Education-wise, they were not receiving that information, because of lack of barrier language.” “I do remember she had a situation where she was asked to sign a lot of documents and she did not know what she was signing. And it was because the social worker had a lot on her plate and she just said, ‘Listen, you can't access, you know, the services that you need for during your pregnancy, if you don't sign these papers.’”
Lack of knowledge of immigrant eligibility for benefits that support health care access	“It's very hard to access prenatal care. The concept that you can apply for insurance [while] pregnant is not well known and people, even, you do know it, a lot of people are afraid to do it, or the barrier of getting online and doing it, it's just too high.” “A lot of people have the assumption I'm immigrant, I don't have no social, I don't have a legal status, I'm not eligible for anything.”

**Facilitators to prenatal care access**	
Flexibility with appointment scheduling process	“I love to see where they can walk into a health center or hospital, and they can get connected easily, not have to call this person that calls that person…”
Ability to bring support persons to prenatal appointments	“We went to the prenatal visits together, and she has been seen, and so I could interpret to her as well, so she didn't feel like she was by herself or on her own. And even though I was there, and the health care provider was using an interpreter over the phone, the fact that I had to rephrase again for her so she will better understand, it made her comfortable actually.”

Despite these challenges, DSPs also discussed workarounds used by their clients that fell into two major themes of facilitators. First, before the pandemic, patients had flexibility with how they made appointments and often preferred to “walk-in” and speak to someone at the front desk directly, rather than trying to communicate over the phone with a scheduler. Second, they were able to bring support people such as family members or CBO staff to appointments, who assisted in filling needed gaps in resources or knowledge, such as interpretation.

### Prenatal care experiences during the ongoing COVID-19 pandemic

*When describing their* pregnant immigrant clients' experiences with prenatal care services after the onset of the COVID-19 pandemic, seven themes emerged that fit into two main categories: barriers to making and attending appointments and barriers to receiving quality care when at appointments (Table 2).

**Table 2.** Heightened Barriers to Quality Prenatal Care Experienced by Direct Service Providers' Immigrant Clients During the Early Months of the COVID-19 Pandemic

Barriers to making and attending appointments	
Heightened fears of deportation due to concurrent widely publicized federal policy changes^a^	“She didn't really want to have any contact with any [medical] professionals because she was worried about her [legal] status and being questioned or getting any authorities involved or anyone knowing too much information that could draw attention to her.”
Increased financial stress due to the pandemic	“Because we do have a lot of clients that are struggling with bills right now and are struggling with rent because of the pandemic, losing jobs, not being able to move around, that's the limitation that we're having.”
Decreased ability to make appointments using previously employed strategies	“It has been challenging even to get an appointment for the client. Most of the issues start from the first step, when we call because the clients cannot walk in already, their working hours were changed during that time, the client could not walk in to do the pregnancy test.”
Heightened childcare barriers to attending prenatal visits	“La única parte que no le gustaba a ella, como tenían hijos a veces pequeños, que tenían que buscar quién les cuidara a los niños porque no podían llevar a nadie más que ella a la cita médica. Y cuando fue momento de ver el género del bebé tampoco podía ir su pareja porque nomás podía ir la embarazada.” (*English translation of above quote) The only part that she didn't like, [because] they had children, sometimes little ones, that they had to find someone that could take care of the kids because they couldn't take anyone besides herself to the medical appointments. And when the moment came to see the gender of the baby, her partner was not able to go because only the pregnant person was allowed to go.*

**Barriers to quality care during appointments**	
Challenges with prenatal care delivered solely through telemedicine	“To tell you the truth, when COVID started, she missed out on a lot of visits because of the virtual, the telemedicine.” “Telemedicine was difficult for the one client that I'm thinking because she didn't have a good phone. So she wasn't able to do the video calls. She could do a phone call, but that was sometimes tricky because maybe her phone was off because of lack of income at the time. So thinking about if we're to go back to, even the quarantine status or anything it was just very difficult for her to access services without me there to use my phone. So internet, phones, that was just very difficult when it came to getting services in that way.”
Care that was not language concordant or culturally sensitive	“When she would go to try to make an appointment to speak with someone at the front desk, unless she brought a translator with her, there was no one there to communicate back and forth with her.” I'm going to give you a really silly example, right? In my husband's culture, he's from [country], it's very typical when a newborn is born to put a bracelet on them. To keep away from the evil eye or things like that. In the United States, you go and you get reprimanded for having a bracelet on them because it's a choking hazard, et cetera, right? So, just explaining those things about having cultural sensitivity around it right now, not being aggressive with when our patients, when we talk to them about those things, taking culture into perspective.
Discriminatory encounters with health care staff when attending visits alone	Yo creo que no importa dónde ellas vayan, que hayan personas empáticas porque hay veces que las enfermeras son rudas o el personal que esté en el “service desk” a veces no son empáticos, no le ayudan a las mamás cuando son primerizas y no saben qué necesitan hacer. *(English translation of above quote)* I think that it doesn't matter where they go, as long as there are empathetic people [staff] because there are times when the nurses are rude or the personnel that are at the service desk are not empathetic. They don't help the first-time mothers who don't know what they need to do.

^a^ For example, the Trump administration's proposed changes to the Public Charge Rule for Inadmissibility went into effect in February of 2020.

Four themes *emerged within the first category. The first theme centered around clients' heightened fears of deportation and adverse immigration sequelae due to concurrent widely publicized federal policy changes.* DSPs discussed how fears of deportation or apprehension were common before the COVID pandemic, but federal policies enacted just before or during the pandemic, such as the public charge rule change of 2019,^25^ resulted in increased hesitancy for many clients. DSPs also discussed how the increased financial stress during the pandemic impacted clients' families' decisions to spend money on health care. While high medical costs existed before COVID, many DSPs reported that COVID-related restrictions resulted in heightened unemployment and widespread inability to pay rent, especially as undocumented immigrant families were ineligible for government-issued stimulus checks.

The third theme in this category centered around DSPs' clients' decreased ability to make appointments using previously employed strategies such as walk-in strategies. This change created more stress for pregnant immigrants, especially those who did not feel comfortable expressing themselves by calling. If clients were able to go in person, there were not always front desk staff or interpreters available as there might have been before the pandemic. Finally, DSPs discussed that their clients suffered from heightened barriers accessing the childcare, which was now required for them to attend, visits as COVID restrictions prohibited other children at appointments.

In the second category of client experiences described by DSPs, clients faced barriers to receiving quality care when at appointments, centered around three themes. First, interviewees described that although the shift to telemedicine during COVID removed some barriers like transportation and childcare, it diminished the quality of care for many pregnant immigrant clients. DSPs reported that some clients missed visits because they simply did not have the appropriate technology to engage in telemedicine during the time when it was the main form of receiving prenatal care in the city. They also struggled with the lack of language accessibility offered by telephone operator menus and virtual visits.

The second and third themes that emerged in this category appeared rooted in the fact that clients could no longer bring support persons with them to appointments due to COVID restrictions. Many DSPs' clients relied on bringing an additional person to their appointments for interpretation and better understanding of what was happening during their prenatal visits. Without the option of a support person, many clients experienced increased language barriers, loss of critical information about their prenatal care, and increased stress at appointments.

Finally, DSPs reported that cultural barriers appeared heightened, perhaps due to increased stressors on health care providers during this novel pandemic. They described that cultural barriers had always existed, but COVID strains on providers may have intensified the effects by limiting provider time in explaining prenatal practices that patients from other countries may not be familiar with. Furthermore, some DSPs told stories about negative experiences their clients experienced in health care settings secondary to rudeness or lack of empathy. DSPs worried these experiences might be related to discrimination their clients experienced because they were presenting for care alone.

### Suggestions for improving access to quality prenatal care

**FIG. 1.**
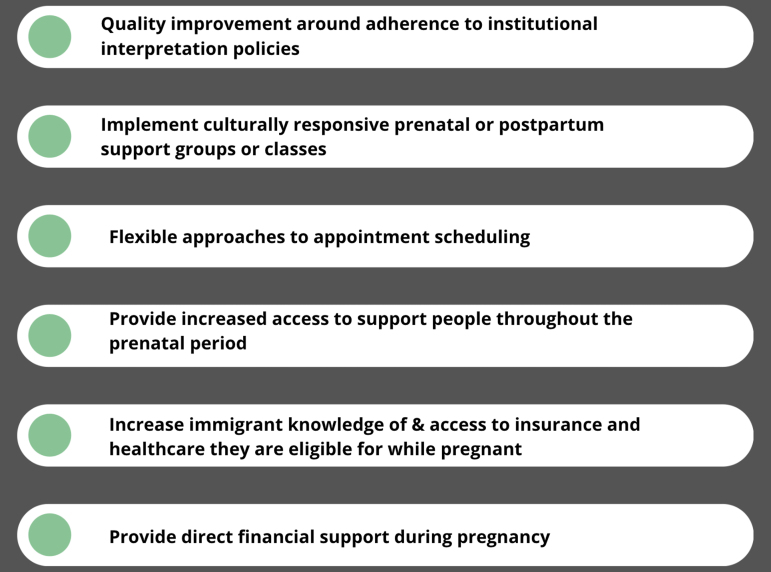
Direct service providers' suggestions for improving access to quality prenatal care for pregnant immigrants during the ongoing COVID-19 pandemic and afterward.

Some DSPs provided suggestions for how to improve access to quality prenatal care for pregnant immigrants at different points during their interviews. However, we also ensured DSPs had the opportunity to offer any further suggestion for improving prenatal care access and quality for pregnant immigrants in Philadelphia at the end of each interview. The themes that arose in this section (Fig. 1) include the need for quality improvement with regard to the care provided in prenatal settings, with DSPs specifically citing clients' experiences with cultural insensitivity and poor adherence to institutional interpretation policies. Second, DSPs suggested reinstituting the flexible options for how patients can schedule appointments, including allowing in-person scheduling at a reception desk. Third, DSPs suggested increasing access to support persons for pregnant immigrants, either through access to personnel like them at CBOs or care navigation.

Many DSPs highlighted the need for patients to be better taught about insurance eligibility and their rights in seeking health care and to receive assistance in signing up for benefits they were eligible for. Finally, DSPs suggested that some clients need direct financial support during pregnancy given the insurmountable nature of their financial challenges. Some suggested that this might look like alternate co-payment policies and assistance in paying for transportation to and from visits. Supplementary Data S2 lists themes and supporting quotes.

## Discussion

This study explored barriers and facilitators to pregnant immigrants' access to quality prenatal care in Philadelphia during the COVID-19 pandemic. Pregnancy and access to health care during this time, are critical to examine from a public health perspective, given the risks to maternal health and the need for regular, repeated preventative visits to optimize the health and wellbeing of a dyad. At the start of the pandemic, experts expected that COVID-19 would exacerbate existing disparities in health care service access among immigrant families, especially as federal COVID-19 relief efforts excluded immigrant families, regardless of documentation status.^10,11^

To date, there are varied findings on the impact of the pandemic on birth outcomes overall in the population,^26^ but clear evidence that racially and ethnically minoritized pregnant people were disproportionately infected by COVID-19.^14,27–30^ Given the further marginalization that occurs for pregnant immigrant people, many of whom are people of color, there is an ongoing need to better understand the lived context driving health outcomes for such groups.

Using a uniquely informed cohort of informants, we found that the pandemic both created and heightened existing barriers for pregnant immigrants' access to prenatal care. It also diminished their ability to use the facilitators to health care access, which had been created to navigate pre-COVID barriers. For instance, patients were no longer allowed to “walk-in” to schedule their visits or bring support persons, who had previously served as cultural or linguistic translators, to their appointments. Although such policies were born out of necessary pandemic precautions to reduce community transmission, they also had unintended consequences of greatly reducing accessibility for those who already had significantly reduced access to language-concordant prenatal care.

Furthermore, the pandemic coincided with the implementation of the expanded federal public charge rule in 2019.^25^ Our informants indicated that pregnant immigrants' hesitancy and fear about being labeled a “public charge” if they engaged with prenatal care services were exacerbated by both pandemic-related job and insurance loss. Although the expanded public charge rule was rescinded by the Biden administration during our study period,^31,32^ DSPs discussed ongoing hesitancy and confusion among their clients related to the combined impact of the pandemic and the media surrounding the expanded public charge rule.

We also found that a major barrier to individuals engaging in health care services was a lack of understanding of legal rights in the prenatal period. This barrier has also been reported by other studies exploring the utilization of pediatric care and the enrollment of government benefit programs among immigrant families during the pandemic.^33–35^ According to 2020 data from the Urban Institute's Well-Being and Basic Needs Survey, more than 1 in 3 (36.1%) adults in families in which one or more members do not have a green card (permanent residence status) reported avoiding a noncash government benefit program they or a family member were eligible for due to concerns about immigration status or enforcement.^35^ This included programs such as Medicaid, the Children's Health Insurance Program, the Supplemental Nutrition Assistance Program, or housing assistance.

To our knowledge, no other qualitative study to date has documented the impact of the ongoing COVID-19 pandemic on the prenatal care experiences of immigrant birthing people in the United States. However, our analyses mirror what other researchers have documented with respect to immigrants' overarching health care experiences in other countries. For instance, Matsuoka et al. recently reported that language barriers to health care and public health information were heightened by the pandemic among their cohort of Southeast Asian immigrants in Japan,^36^ as was reported by the DSPs in our study. A qualitative study of the health care experiences of refugees and immigrants in Canada also highlighted pandemic-heightened challenges to making health care appointments similar to what our DSPs reported pregnant immigrants in Philadelphia experienced.^37^

Our work illuminates several important opportunities for local policymakers and health care institutions to optimize prenatal care for immigrants, both during this ongoing pandemic and future public health emergencies. First, when designing policies around support persons during maternal care delivery, health care administrators may consider balancing infection control concerns with the unique vulnerabilities of pregnant immigrants with limited English proficiency.

Administrators could also consult with DSPs to understand the unique implications of policies that alter health system accessibility for immigrant populations. Second, ongoing efforts are needed to better disseminate prenatal care eligibility information among immigrant communities. Given that immigrant-focused CBOs often have deep, trusted ties to immigrant communities, health care institutions and public health agencies could create formal, compensated consultant relationships with such CBOs that allow for more targeted and effective dissemination of key information and resources regarding how to access and navigate health care to immigrant people. This would amplify and equitably compensate the unfunded or underfunded work DSPs within CBOs already do in immigrant communities.

Our study also highlights the relationship between barriers to affordable childcare and optimal health care utilization. Our participants reported lack of childcare options as a major barrier to attending prenatal care visits, especially when COVID restrictions prohibited other children at appointments. Difficulties accessing affordable childcare have been shown to be a prominent issue among immigrant communities even before the pandemic,^38^ but it is highly likely the pandemic exacerbated this challenge. Poor childcare accessibility is not restricted to pregnant immigrants. However, our findings indicate the need for continued attention toward mitigating these challenges for pregnant patients, especially those who are at higher risk of socioeconomic barriers like certain immigrant communities.

Finally, this study offers an evidence base on which to explore health system strategies to improve engagement and support for pregnant immigrant clients, including creating language-concordant peer counseling opportunities, exploring innovative group care delivery models for immigrants, or partnering with CBOs that can offer access to language-concordant doulas. In addition, immigrant access to nurse home visiting programs could be bolstered, given the evidence linking such prenatal nurse home visitation to positive child health outcomes, as well as the fact that immigrants continue to be underserved by such programs.^39^ Many of these interventions would benefit from the integration of DSPs into the maternal care delivery system, which will require reimbursement innovation within payer systems.

### Limitations

Our study is limited by generalizability, given the small sample of DSPs in the Philadelphia region. While the experiences of our DSPs' clients are likely representative of many pregnant immigrants in our region, findings may be different among pregnant immigrants who are less connected to or have not sought out the community-based social services that our DSPs represent. In addition, we limited our sample of interview participants to DSPs at CBOs rather than with immigrants who were pregnant during COVID-19. Thus, we relied on DSPs' memories of client-reported experiences, which introduce the possibility of recall bias. Finally, it is possible that clients only discussed barriers and challenges to prenatal care that they felt were relevant to their relationship with the DSP, and as such, our participants' insight presents only a limited picture.

However, our decision to interview DSPs was intentional for two main reasons. First, we sought to protect the mental well-being and privacy of a group whose vulnerability continues to be heightened during this ongoing pandemic and mitigate any immigration status-related fear among potential participants. Second, we felt that interviewing DSPs might allow us to avoid any social desirability bias that might arise during interviews with immigrant participants. Finally, interviewing DSPs who work closely with many individuals in the target population allowed us access to narratives that summarize the experiences of many clients in our region.

## Conclusions

This study describing barriers and facilitators to pregnant immigrants' access to quality prenatal care services during COVID-19 adds to the growing body of research on the drivers of health inequities associated with the global pandemic. Our study highlights that health care and social service providers involved in maternal care delivery can and should play important roles in supporting the health and well-being of pregnant and postpartum immigrant individuals, especially those for whom access to care is limited.

Our findings also have critical policy implications that can guide structural solutions such as optimizing prenatal care access among systematically disadvantaged groups like pregnant immigrants in the United States. For instance, future work could use the community-derived insight from this study to implement and inform strategies such as alternative care delivery models and the payment innovation needed to fund such models. In this way, health care providers and policymakers can align efforts to promote health equity among immigrant birthing people and their families during and beyond this pandemic.

## Supplementary Material

Supplemental data

Supplemental data

## Abbreviations Used

CBOs: community-based organizations
DSPs: direct service providers
FQHCs: Federally Qualified Health Centers

## References

[1] Heaman M, Bayrampour H, Kingston D, et al. Migrant women's utilization of prenatal care: A systematic review. Matern Child Heal J 2012;17(5):816–836; doi: 10.1007/S10995-012-1058-Z 22714797

[2] Korinek K, Smith KR. Prenatal care among immigrant and racial-ethnic minority women in a new immigrant destination: Exploring the impact of immigrant legal status. Soc Sci Med 2011;72(10):1695; doi: 10.1016/J.SOCSCIMED.2011.02.046 21530038 PMC6125157

[3] Maru S, Glenn L, Belfon K, et al. Utilization of maternal health care among immigrant mothers in New York City, 2016–2018. J Urban Health 2021;98(6):711; doi: 10.1007/S11524-021-00584-5 34811699 PMC8688674

[4] Pew Research Center. U.S. Birth Rate Falls to a Record Low; Decline Is Greatest Among Immigrants. n.d. Available from: https://www.pewresearch.org/social-trends/2012/11/29/u-s-birth-rate-falls-to-a-record-low-decline-is-greatest-among-immigrants/ [Last accessed: November 15, 2022].

[5] Fabi R. Prenatal care for undocumented immigrants: implications for policy, practice, and ethics. Popul Heal Res Br Ser 2020. Available from: https://www.maxwell.syr.edu/research/lerner-center/population-health-research-brief-series/article/prenatal-care-for-undocumented-immigrants-implications-for-policy-practice-and-ethics [Last accessed: December 16, 2022].

[6] Castañeda H, Holmes SM, Madrigal DS, et al. Immigration as a social determinant of health. Annu Rev Public Health 2015;36:375–392; doi: 10.1146/ANNUREV-PUBLHEALTH-032013-182419 25494053

[7] Pillai D, Artiga S. 2022 Changes to the Public Charge Inadmissibility Rule and the Implications for Health Care. 2022. Available from: https://www.kff.org/racial-equity-and-health-policy/issue-brief/2022-changes-to-the-public-charge-inadmissibility-rule-and-the-implications-for-health-care/ [Last accessed: March 23, 2023].

[8] Nwadiuko J, German J, Chapla K, et al. Changes in health care use among undocumented patients, 2014–2018. JAMA Netw Open 2021;4(3):e210763; doi: 10.1001/JAMANETWORKOPEN.2021.0763 33666662 PMC7936260

[9] Friedman AS, Venkataramani AS. Chilling effects: US immigration enforcement and health care seeking among hispanic adults. Health Aff (Millwood) 2021;40(7):1056–1065; doi: 10.1377/HLTHAFF.2020.02356 34228522

[10] Fabi RE, Ludmir J. Pregnancy, pandemics, and public health policy: The disparate impact of COVID-19 on pregnant immigrants. Womens Heal Issues 2021;31(3):195–197; doi: 10.1016/j.whi.2020.12.001 PMC7836935 33461870

[11] Clark E, Fredricksid K, Woc-Colburn L, et al. Disproportionate impact of the COVID-19 pandemic on immigrant communities in the United States. PLoS Negl Trop Dis 2020;14(7):e0008484; doi: 10.1371/JOURNAL.PNTD.0008484 32658925 PMC7357736

[12] Cholera R, Falusi OO, Linton JM. Sheltering in place in a xenophobic climate: COVID-19 and children in immigrant families. Pediatrics 2020;146(1):e20201094; doi: 10.1542/PEDS.2020-1094/37023 32345687

[13] Ellington S. COVID-19 in Pregnant People and Infants Ages 0-5 Months. n.d. Available from: https://www.cdc.gov/vaccines/acip/meetings/downloads/slides-2022-10-19-20/02-03-04-covid-ellington-kharbanda-olson-fleming-dutra-508.pdf [Last accessed: December 16, 2022].

[14] Montoya-Williams D, Mullin AM, Handley SC, et al. Disparities in SARS-CoV-2 positivity among pregnant patients with limited English proficiency. J Perinatol 2021;41(10):2564–2565; doi: 10.1038/s41372-021-01148-w 34497337 PMC8425017

[15] Burris HH, Mullin AM, Dhudasia MB, et al. Neighborhood characteristics and racial disparities in severe acute respiratory syndrome coronavirus 2 (SARS-CoV-2) seropositivity in pregnancy. Obstet Gynecol 2022;139(6):1018–1026; doi: 10.1097/AOG.0000000000004791 35675599 PMC9180815

[16] Bloemraad I, Terriquez V. Cultures of engagement: The organizational foundations of advancing health in immigrant and low-income communities of color. Soc Sci Med 2016;165:214; doi: 10.1016/J.SOCSCIMED.2016.02.003 26898114 PMC5012884

[17] Roth BJ, Gonzales RG, Lesniewski J. Building a stronger safety net: Local organizations and the challenges of serving immigrants in the suburbs. Hum Serv Organ Manag Leadersh Gov 2015;39(4):348–361; doi: 10.1080/23303131.2015.1050143

[18] Fox KS, Kahn-Troster S. Advancing health equity in health care coverage: A public-private partnership to engage underserved communities in medicaid expansion. J Health Care Poor Underserved 2022;33(4S):44–60; doi: 10.1353/HPU.2022.0158 36533458

[19] Salib Y, Amodei J, Sanchez C, et al. The COVID-19 vaccination experience of non-English speaking immigrant and refugee communities of color: A community co-created study. Community Health Equity Res Policy 2022; [Epub ahead of print]; doi: 10.1177/2752535X221133140 PMC9597283 36283968

[20] Wiley Z, Khalil L, Lewis K, et al. A framework for inspiring COVID-19 vaccine confidence in African American and Latino Communities. Vaccines 2022;10(8):1319; doi: 10.3390/VACCINES10081319 36016207 PMC9416715

[21] Laguna-Torres A, Velosa L, Barreto A, et al. Overcoming barriers to the recruitment of immigrant Hispanic people in perinatal research. Health Serv Res 2023;58(3):543–548; doi: 10.1111/1475-6773.14127 36600513 PMC10154151

[22] Neiger R. Long-term effects of pregnancy complications on maternal health: A review. J Clin Med 2017;6(8):76; doi: 10.3390/JCM6080076 28749442 PMC5575578

[23] Glazer KB, Zeitlin J, Howell EA. Intertwined disparities: Applying the maternal-infant dyad lens to advance perinatal health equity. Semin Perinatol 2021;45(4):151410; doi: 10.1016/J.SEMPERI.2021.151410 33865629 PMC8184592

[24] Singer A, Vitiello D, Katz M, et al. Recent Immigration to Philadelphia: Regional change in a re-emerging gateway. Brookings Inst 2008;November:1–40.

[25] Homeland Security Department. Inadmissibility on Public Charge Grounds. 2019. Available from: https://www.federalregister.gov/documents/2019/08/14/2019-17142/inadmissibility-on-public-charge-grounds [Last accessed: December 16, 2022].

[26] Chmielewska B, Barratt I, Townsend R, et al. Effects of the COVID-19 pandemic on maternal and perinatal outcomes: a systematic review and meta-analysis. Lancet Glob Heal 2021;9(6):e759–e772; doi: 10.1016/S2214-109X(21)00079-6 PMC8012052 33811827

[27] Janevic T, Glazer KB, Vieira L, et al. Racial/ethnic disparities in very preterm birth and preterm birth before and during the COVID-19 pandemic. JAMA Netw Open 2021;4(3):e211816; doi: 10.1001/JAMANETWORKOPEN.2021.1816 33729505 PMC7970336

[28] Barrero-Castillero A, Beam KS, Bernardini LB, et al. COVID-19: Neonatal-perinatal perspectives. J Perinatol 2021;41(5):940–951; doi: 10.1038/S41372-020-00874-X 33293665 PMC7721617

[29] Chae SY, Bhattacharyya A, Mendoza R. COVID-19 in pregnancy: A current review of global cases. Obstet Gynecol Surv 2021;76(8):504–513; doi: 10.1097/OGX.0000000000000925 34449853

[30] Son M, Gallagher K, Lo JY, et al. Coronavirus disease 2019 (COVID-19) pandemic and pregnancy outcomes in a U.S. population. Obstet Gynecol 2021;138(4):542–551; doi: 10.1097/AOG.0000000000004547 34433180 PMC8454282

[31] KPMG Global. United States—DHS Public Charge Rule Rescinded. 2021. Available from: https://home.kpmg/xx/en/home/insights/2021/03/flash-alert-2021-087.html [Last accessed: December 16, 2022].

[32] U.S. Citizenship and Immigration Services. Public Charge. 2022. Available from: https://www.uscis.gov/public-charge [Last accessed: December 16, 2022].

[33] Raphael JL, Beers LS, Perrin JM, et al. Public charge: An expanding challenge to child health care policy. Acad Pediatr 2020;20(1):6–8; doi: 10.1016/j.acap.2019.09.001 31521776

[34] Galletly CL, Barreras JL, Lechuga J, et al. US public charge policy and Latinx immigrants' thoughts about health and healthcare utilization. Ethn Health 2023;28(1):96–113; doi: 10.1080/13557858.2022.2027879 35166623 PMC9376191

[35] Urban Institute. Immigrant Families Continued Avoiding the Safety Net during the COVID-19 Crisis. n.d. Available from: https://www.urban.org/research/publication/immigrant-families-continued-avoiding-safety-net-during-covid-19-crisis [Last accessed: November 15, 2022].

[36] Matsuoka S, Kharel M, Koto-Shimada K, et al. Access to Health-Related Information, Health Services, and Welfare Services among South and Southeast Asian Immigrants in Japan: A Qualitative Study. Int J Environ Res Public Health 2022;19(19):12234; doi: 10.3390/ijerph191912234 36231533 PMC9566169

[37] Leung D, Lee C, Wang AH, et al. Immigrants' and refugees' experiences of access to health and social services during the COVID-19 pandemic in Toronto, Canada. J Health Serv Res Policy 2023;28(1):34–41; doi: 10.1177/13558196221109148 35971256 PMC9382571

[38] Sandstrom H, Gelatt J. Child care choices of low-income, immigrant families with young children: Findings from the National Survey of Early Care and Education. 2017. Available from: https://www.immigrationresearch.org/system/files/child-care-choices-of-low-income-immigrant-families-with-young-children.pdf [Last accessed: March 23, 2023].

[39] Park M, Katsiaficas C. Leveraging the potential of home visiting programs to serve immigrant and dual language learner families. Policy Brief. 2019. Available from: https://www.migrationpolicy.org/research/home-visiting-immigrant-dual-language-learner-families [Last accessed: March 23, 2023].

