# Supplementary material for: Exploring Prenatal Care Quality and Access During the COVID-19 Pandemic Among Pregnant Immigrants in Philadelphia Through the Lens of Community-Based Organizations
Source: Womens Health Rep (New Rochelle). 2023 May 17;4(1):241–50. doi: 10.1089/whr.2022.0112 (PMC10240328; doi:10.1089/whr.2022.0112)
Supplement: Supplemental data [file Supp_DataS1.docx]

**SUPPLEMENT 1: SEMI STRUCTURED INTERVIEW GUIDE**

**Questions formed a guide but participants were allowed to lead the conversations or focus on some questions but not others. Not all participants answered all questions.**

**Part I: General**

- *To start off, would you describe the work that you do with this organization?* What kind of services do you provide (literacy, health, service navigation, prenatal, parenting, etc.)
- Would you describe the community you serve? (local area, language, countries of origin, prenatal/parenting women, number of families served)
  - Do you serve immigrant families?
  - How would you describe the primary immigrant communities your clients come from?
  - How do you refer to clients that you work with? (Community members, individuals, etc.)
- **Systems Level**
  - Are there health services that your organization partners with?
    - Delivery hospitals, Prenatal services, Federally Qualified Health Centers (FQHCs), other?
    - If yes,
      - Do you have any shared meetings or activities with any health service providers?
      - Do you have any data-sharing agreements with any health service providers?
      - Do you provide or receive any consultation with any health service providers?
  - Do you provide or receive any referrals or warm handoffs with any health service providers?
  - Do you work with any other prenatal or pregnancy-related service providers (i.e. community doula programs, home visiting programs, etc.)
  - Have any of these relationships changed since COVID?
- How else has COVID impacted your operations?

**Part II: Accessing Quality Prenatal Care- Before COVID**

*I’d like to start off by having you to think back to before COVID. These questions are specifically focused on immigrant clients or differences between immigrant clients and non-immigrant clients.*

- Could you describe your clients’ typical experiences accessing quality prenatal care services?
  - What services do your clients typically access while pregnant? Quality?
  - Where do clients typically receive prenatal health care?
  - What are your perceptions of unmet client needs? Are there typically gaps in services that your clients receive? Quality issues?
  - How do your clients typically feel about pregnancy and prenatal care? Are there typical anxieties among your clients surrounding pregnancy?
- **Case Narrative**

*Thinking about the last pregnant client you worked with before COVID…*

- - What was her experience with pregnancy? How did she feel about her pregnancy?
  - What was her experience accessing services (healthcare or otherwise) during this time?
  - Do you have a sense of the quality of these services or her interactions with medical providers?
  - Do you know if she had any particular medical issues that impacted her pregnancy/her child?
  - Did she or her family face any particular hardships while pregnant?
    - Were any of these hardships related to family documentation status?
    - Were any of these hardships related to medical insurance coverage?
  - Were there any people, services, or resources that made pregnancy/accessing prenatal care easier for your clients?
  - Does her experience seem typical for all your pregnant clients? How is this different or similar to the ‘typical’ experience?

**Part III: Accessing Prenatal Care- During COVID**

Now we are going to talk about immigrant clients’ prenatal experiences since COVID began (March 2020).

- **Case Narrative**

*Thinking about the last pregnant client you worked with during COVID…*

- - What was her experience with pregnancy? How did she feel about her pregnancy?
    - How far into COVID was your client pregnant/delivering the baby and how do you think this affected her experience?
  - What was her experience accessing services (healthcare or otherwise) during this time?
  - Do you have a sense of the quality of these services or her interactions with medical providers?
  - Could you describe her experience transitioning to telemedicine (if anything moved to telemedicine)?
  - Could you describe her experiences with in-person services?
  - How do you think her experience was affected by COVID, if at all?
  - Do you know if she had any particular medical issues that impacted her pregnancy/her child?
  - Did she or her family face any particular hardships while pregnant?
    - Were any of these hardships related to family documentation status?
    - Were any of these hardships related to medical insurance coverage?
  - Were there any people, services, or resources that made pregnancy/accessing prenatal care easier for your clients?
  - Does her experience seem typical for all of your pregnant clients? How is this different or similar to the ‘typical’ experience?
- *Across all clients, have you seen any differences in access to prenatal services and prenatal service quality by clients’…*
  - How far into COVID pregnant/delivering?
  - Primary language? English language proficiency? Literacy in any language?
  - Other medical complications/conditions or medical problems with the pregnancy or child’s birth?
  - Client or family documentation status?
  - Insurance status or Medicaid enrollment?

**Part IV: Meeting Client Needs**

*We’re now going to ask you to brainstorm possible supports for your clients.*

- What is the ideal pregnancy experience for the women you work with?
- As COVID continues or comes to an end, what do you think would make it easier for pregnant immigrant women you work with to access prenatal care?
  - What supports do your clients need to better navigate services and systems during their pregnancies?
  - What provider or institutional policies would improve the quality of prenatal care for your clients?
  - What other resources would be useful for your clients? What additional supports are needed for your clients with medical complexities or adverse birth outcomes?
  - Are there additional systems-level connections your organization needs to support your pregnant clients?
    - Health care service providers, or otherwise?
    - Data sharing agreement, shared meetings or activities, consultation, referrals/warm handoffs, etc.
- After COVID, if things return to more normal (more face-to-face interactions, less risk of transmission), what would make it easier for pregnant immigrant women you work with to access quality prenatal care? Are these the same barriers?
  - How could medical providers better support clients who did not receive adequate prenatal care during this time?
  - What supports were helpful during COVID that should continue after?
