# Supplementary material for: Exploring Prenatal Care Quality and Access During the COVID-19 Pandemic Among Pregnant Immigrants in Philadelphia Through the Lens of Community-Based Organizations
Source: Womens Health Rep (New Rochelle). 2023 May 17;4(1):241–50. doi: 10.1089/whr.2022.0112 (PMC10240328; doi:10.1089/whr.2022.0112)
Supplement: Supplemental data [file Supp_DataS2.docx]

**Supplement 2: Quotes supporting DSP’s Suggestions for Improving Access to Quality Prenatal Care For Pregnant Immigrants**

| **Suggestion** | **Quotation** |
| --- | --- |
| Quality improvement with regards to adherence to institutional interpreter policies | “Alot of the practices rely on people to take their own interpreters. So you’re, you’re, you’re, you’re bound by your friends or sister or partner’s schedule whether or not you can even make those appointments. So the missed appointment rate is high.”  “The health facility doesn’t always have an interpreter available online to help out when they call the clients.” |
| Implementing culturally responsive prenatal or postpartum support groups or classes | “First, to prenatal classes that are culturally sensitive that are in their own language and also get connected with programs. I know there's a breastfeeding one where it's like, if you're having a hard time breastfeeding, you can call a mom that's in this group and you can tell her your woes and she can walk you through it. So that, for any stage of pregnancy or child rearing, I think connecting parents with parents is a great way to create support systems for raising our kids.” |
| Flexible approaches to appointment scheduling | “So I do believe that first of all the appointment process need to be made easier, like to be able to find [an appointment]…If we can get the chance to be able to talk over the phone to someone and to get the appointment done, instead of having to call the clients, it will be great. |
| Providing increased access to support people throughout the prenatal period | “I think [support persons] should be more accessible for them…like more services to be offered for a pregnant woman and a person that, a social worker for example, that is all the time from the beginning of the pregnancy, supporting this family until the end of the pregnancy. And after the baby's delivery, keep going a couple months until that plan is already set up and the baby's okay too.”  “I think that the model that nurse family partnership has, or you have somebody who will count. I mean, I think that works very well.” |
| Efforts to increase immigrants’ awareness of potential rights’ to insurance and healthcare while pregnant and assist patients with accessing benefits | “Yo creo que el que le tengan quizás un paquete bien claro de todos los servicios que [la ciudad] de Filadelfia que pueden conseguir para sus niños gratuitos, ya que ellos son inmigrantes. Es que hay muchos temores en ellos.”  (*English translation of above quote) “I believe that perhaps they could have a clear package of services that the [city] of Philadelphia can get for their children for free since they are immigrants. They have a lot of fear [about using services].”*  “I think it's just bringing attention to students or patients or clients that they have rights, even if they're undocumented, they have rights. |
| Provision of direct financial support during pregnancy | “Even maybe having some tokens available or funds available for Uber rides, things of that nature to just help with that.”  [It would be good for clients] “to not worry about a big bill coming up, because you're giving birth. To not [have] to pay for co-payments.... Sometimes, you are going through so much things, and you don't have out of pocket $5 to pay. |
